# Electronic Structure and Magnetism of Mn-Doped ZnO Nanowires

**DOI:** 10.3390/nano5020885

**Published:** 2015-05-27

**Authors:** Fuchun Zhang, Dandan Chao, Hongwei Cui, Weihu Zhang, Weibin Zhang

**Affiliations:** 1 College of Physics and Electronic Information, Yanan University, Yanan 716000, China; E-Mails: yadxzfc@yau.edu.cn (F.Z.); yadxddc@163.com (D.C.); hweicui001@163.com (H.C.); ydzwh@163.com (W.Z.); wbzhang@dongguk.edu (W.Z.); 2 Department of Physics, Dongguk University, Seoul 100-715, Korea

**Keywords:** ZnO, dilute magnetic semiconductors, nanowire, density functional theory, magnetic property

## Abstract

The geometric structures, electronic and magnetic properties of Mn-doped ZnO nanowires were investigated using density functional theory. The results indicated that all the calculated energy differences were negative, and the energy of the ground state was 0.229 eV lower than ferromagnetic coupling, which show higher stability in antiferromagnetic coupling. The calculated results indicated that obvious spin splitting phenomenon occurred near the Femi level. The Zn atoms on the inner layer of ZnO nanowires are easily substituted by Mn atoms along the [0001] direction. It was also shown that the Mn^2+^-O^2−^-Mn^2+^ magnetic coupling formed by intermediate O atom was proved to be caused by orbital hybridization between Mn *3d* and O *2p* states. The magnetic moments were mainly attributed to the unpaired Mn *3d* orbitals, but not relevant with doping position of Mn atoms. Moreover, the optical properties of Mn-doped ZnO nanowires exhibited a novel blue-shifted optical absorption and enhanced ultraviolet-light emission. The above results show that the Mn-doped ZnO nanowires are a new type of magneto-optical materials with great promise.

## 1. Introduction

Dilute magnetic semiconductors (DMSs) have attracted much attention due to their unique potential usage of both charge and spin of freedom of carriers in magneto-optical, magneto-electrical, and magneto-transport devices [1,2,3,4], Especially, oxide-diluted magnetic semiconductors (such as ZnO) have shown excellent piezoelectric and photoelectric properties, and great potential applications in spintronic devices [5,6,7]. Moreover, theoretical and experimental research has predicted Curie temperatures (*T_C_*) above room temperature, high solubility of magnetic ions, and transparency for visible light in ZnO-based DMSs [8,9]. In recent years, great progress has been achieved in one-dimensional (1D) ZnO-based DMSs. Chang *et al.* [10] prepared Zn_1__−*x*_Mn*_x_*O nanowires (NWs) at 500 °C with a high doped content of 13%. Philipose *et al.* [11] also successfully prepared Zn_1__−*x*_Mn*_x_*O NWs, and the samples showed stable ferromagnetic (FM) and ultraviolet emission properties. Wang *et al.* [12] prepared Zn_1__−*x*_Mn*_x_*O NWs and no second phase was observed in the Mn-doped ZnO NWs. Theoreticians have found ZnO to be an excellent candidate host semiconductor for high-*T_C_* ferromagnetism. Dietl *et al.* [2] made theoretical predictions about the higher Curie temperature of Mn-doped ZnO, Sato *et al.* [13] revealed the magnetic properties of *3d* TMs-doped ZnO by using the local density approximation (LDA). Marcel *et al.* [14] investigated the ferromagnetic (FM) and antiferromagnetic (AFM) properties of *3d* TMs-doped ZnO. Our previous studies have shown that potential FM ground states are more stable in V-doped and Fe-doped ZnO NWs [15,16], and have obvious half-metallic properties in Fe-doped ZnO NWs [16]. In addition, He *et al.* [17] have reported on the electronic and magnetic properties of Mn-doped ZnO nanotubes using density functional theory (DFT) with the generalized gradient approximation (GGA). However, many problems in the research of ZnO-based DMSs still remain both scarce and controversial regarding the influence of impurities on the electronic, optical, and magnetic properties. The magnetic coupling mechanism and origin of the ferromagnetism is still not clear, and experimental observations demonstrate controversial results about optical phenomenon in the optical absorption with regards to Mn-doped ZnO NWs. In order to clarify the effect of Mn doping on the electronic, optical, and magnetic properties of ZnO NW, it is necessary to perform first-principles methods based on DFT.

In this paper, the geometric structures, and electronic and magnetic properties of Mn-doped ZnO NWs were systematically investigated by first-principles methods, based on DFT. The applicable methods to adjust and control oxide DMSs were obtained by analysis of the magnetic coupling mechanism of Mn-doped ZnO NWs. We systematically study mechanisms of FM and AFM coupling on the electronic, optical, and magnetic properties. The main results provide theoretical guidance for preparing ZnO-based DMS materials of high quality and high-*T_C_.*

## 2. Theoretical Models and Calculated Methods

**Figure 1:**
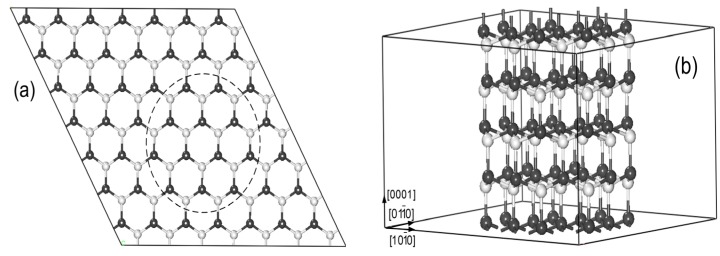
(**a**) The top view of the 7 × 7 × 2 ZnO supercell structure; (**b**) Zn_48_O_48_ nanowires (NWs) supercell along the [0001] direction (The white and black spheres represent O and Zn atoms).

**Figure 2:**
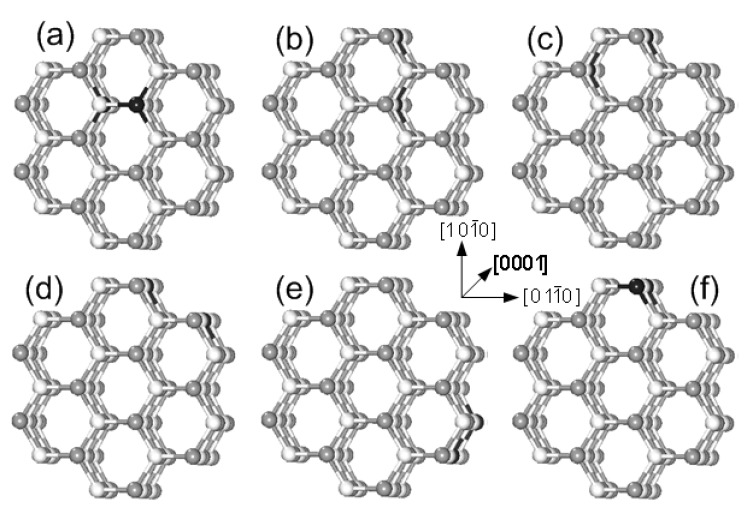
The structures of Mn-doped ZnO NWs. (**a**) Configuration I; (**b**) Configuration II; (**c**) Configuration III; (**d**) Configuration IV; (**e**) Configuration V; (**f**) Configuration VI. (The white, gray, and black spheres represent O, Zn, and Mn atoms, respectively).

The models for Mn-doped ZnO NWs are generated from a wurtzite ZnO (7 × 7 × 2) supercell structure along the [0001] direction (see Figure 1a). All atoms within the dashed line remained to build up the ZnO NW structure in Figure 1b. Our NW model contains 96 atoms (Zn46Mn2O48). The vacuum region of ZnO NWs along [$01\bar{1}0$] and [$10\bar{1}0$] is 15 Å to avoid the effects of the interaction between Mn-doped ZnO NWs on the results, and we select the periodical structure along [0001] direction, as shown in Figure 1b. To investigate the magnetic coupling mechanism between the two Mn atoms, we replace two of the Zn atoms with Mn in ZnO NWs. The six possible magnetic coupling models are as shown in Figure 2, and Mn doping concentration is at 4.2%.

The calculations were carried out by using the first principles methods based on DFT, additionally, the Vienna *Ab initio* Simulation Package (VASP) was applied in our work [18]. The detailed parameters were set as follows, the exchange-correlation function was calculated based on GGA in the form of the Perdew-Burke-Ernzher (PBE) [19], the valence electronic configuration of O, Zn and Mn atoms were 2*s*^2^2*p*^4^, 4*s*^2^3*d*^10^ and 4*s*^2^3*d*^5^, respectively, the cut-off energy of plane wave was set at 420 eV, and the convergence in energy and force was less than 1 × 10^−5^ eV and 10^−3^ eV/Å, respectively, and the stress on the cell was less than 0.01 GPa. The Brillouin zone was sampled with a mesh of 1 × 1 × 16 k points, generated by the Monkhorst-Pack scheme.

## 3. Results and Discussion

**Figure 3:**
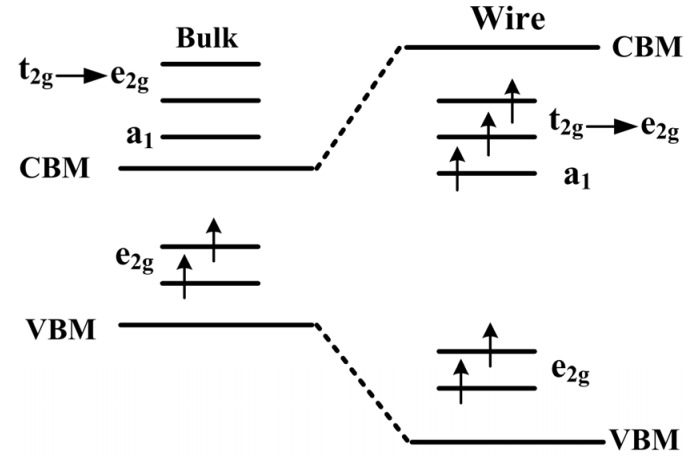
The schematic plot of *3d* level splitting in the ZnO bulk and NW.

To verify the geometric structure, and electronic and magnetic properties of the Mn-doped ZnO NWs, the geometric structures and electronic properties of pure Zn_48_O_48_ NW were calculated under full geometry relaxation. The total energy of the optimized ZnO NW was found to be 6.371 eV lower than that of the unoptimized one. The optimized Zn–O bond length along the [0001] direction was 1.899 Å on the outermost surface layer, which was 4.67% shorter than that of bulk ZnO. The optimized Zn–O bond lengths were 1.969 Å and 1.978 Å on the inner-layer NWs, and 1.15% and 0.7% shorter than that of bulk ZnO, respectively. The optimized Zn–O bond lengths along the [$01\bar{1}0$] direction were between 1.955 Å and 1.962 Å on the outermost surface layer, their corresponding bond angles of ∠O–Zn–O (108.04°) and ∠Zn–O–Zn (108.04°) changed into ∠O–Zn–O (113.76°) and ∠Zn–O–Zn (105.81°). In addition, it was observed from Figure 3a that the total density of states (DOS) for the spin-up and spin-down were identical, namely, pure ZnO NW is a nonmagnetic material. The calculated band gap was 1.86 eV, which was significantly larger than that of our calculated bulk ZnO (0.97 eV).

Table 1 illustrates the magnetic coupling modes, geometric parameters, energy, bond lengths, and magnetic moments of Mn-doped ZnO NW, in which Δ*E* (Δ*E = E_AFM_* − *E_FM_*) denotes energy difference after optimized treatments, Δ*E*_0_ (Δ*E = E_AFM_* − *E_FM_*) denotes the unoptimized energy difference, and the relative energy Δε denotes the energy difference corresponding to the ground state configuration. As shown in Table 1, the Δ*E* of Mn-doped ZnO NWs are all negative, which indicates that the AFM states are more stable than FM states. The configuration V corresponds to the ground state of Mn-doped ZnO nanowires, and its energy is lower than FM state energy by 0.229 eV. In this configuration, the two Mn atoms reside in the inner layer sites along the [0001] direction. As for configuration VI, the energy difference Δ*E* is only −0.007 eV, which indicates that AFM coupling is slightly more stable than FM coupling. In order to further investigate AFM interactions, we note that the corresponding energies of FM and AFM states are almost same as shown in Table 1 (*ΔE*=-0.007 eV), and the AFM coupling is weak with the increase of Mn–Mn bond length for configuration VI (*d*_Mn–Mn_ = 5.205 Å). Hence, it is prone to forming a paramagnetic state and spin-glass state. It is indicated that the Mn–O bond length may play a more important role than the Mn–Mn bond length in predicting the magnetic coupling, which suggests that the range of magnetic interactions between the two Mn atoms is rather short and independent of the Mn–Mn distance. While configurations II, III, and IV are in an AFM state for Mn-doped ZnO nanowires along other directions. Our calculated results are consistent with previously reported results [11,20,21,22]. Moreover, all magnetic moments of Mn atoms are 3.947–4.193 μ_B_ and mainly originate from *3d* electrons of Mn atoms. Oxygen atoms also show AFM spin polarization in nearest-neighbor Mn atoms, which produce a weak magnetic moments of −0.061–−0.089 μ_B_. As for the relaxed geometric structure, the calculated results show that the Mn–O and Mn–Mn bond lengths are 1%–3% shorter than that of an unrelaxed one. The outer layer of ZnO NWs shows more intense relaxation than the inner one, therefore presenting high surface effects and quantum size effects.

**Table 1:** Magnetic coupling modes, energy, bond lengths, and magnetic moments.

Modes	Δ*E* (eV)	Δ*E*_0_ (eV)	Δε (eV)	Coupling	*d*_Mn__–__O_ (Å)	*d*_Mn__–__Mn_ (Å)	Mn_1_ (μ_B_)	Mn_2_ (μ_B_)	O (μ_B_)
I	−0.302	−0.052	0.721	AFM	1.914	3.134	4.171	−4.176	−0.061
II	−0.219	−0.053	0.208	AFM	1.819	3.111	4.134	−4.070	−0.069
I	−0.144	−0.047	0.405	AFM	1.916	3.060	3.947	−4.113	−0.074
IV	−0.139	−0.095	0.561	AFM	1.816	3.264	4.021	−4.104	−0.071
V	−0.229	−0.061	0.000	AFM	1.839	2.694	4.078	−4.067	−0.070
VI	−0.007	−0.003	0.117	AFM	1.823	5.205	4.193	−4.021	−0.089

**Figure 4:**
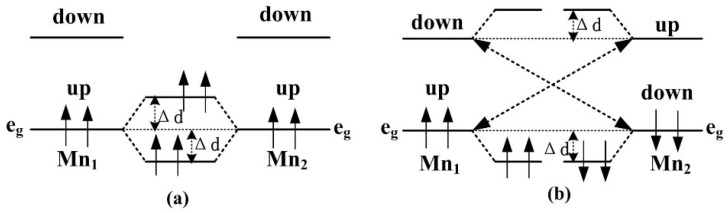
The ferromagnetic (FM) and antiferromagnetic (AFM) mechanisms of the Mn-doped ZnO NW. (**a**) FM coupling; (**b**) AFM coupling.

To further study the origin of magnetic coupling between two Mn atoms, Figure 3 displays the schematic of splitting 3*d* level occupation in bulk ZnO and NW. For Mn-doped ZnO NW, the positions of Zn^2+^ ions are replaced by Mn^2+^ ions, and an Mn^2+^ (*d*^5^ configuration) ion has an tetrahedral structure in a crystalline field. The 3*d* states of an Mn atom split into one upper, triply degenerate $t_{2g}$ state (*d_xy_*, *d_yz_*, *d_xz_*) and one lower, doubly degenerate $e_{g}$ state ($d_{z^{2}-r^{2}}$, $d_{x^{2}-y^{2}}$). However, the space group of ZnO with a wurtzite structure degrades from a high symmetric *T_d_* to a *C*_3*v*_ group in the NW, the $t_{2g}$ states are no longer the original triply degenerate, but further divide into one doubly degenerate $e_{2g}$ state and one singly *a*_1_ state. In particular, O 2*p* states also splits into an $e_{g}$ state and an *a*_1_ state, due to the high symmetry of *T*_2_ in O 2*p* orbital. Magnetic coupling between O 2*p* and Mn 3*d* states cause localization characteristics of the $e_{g}$ states owing to the same symmetry. Figure 4 shows the FM and AFM mechanisms of the Mn-doped ZnO NW. According to the Hund rules and the Pauli exclusion principle, the two lower $e_{g}$ levels located in the gap are fully occupied in the upper-spin bands, and pushed upward and downward by the same Δ*d* to form an FM coupling when there is no energy input, as shown in Figure 3 and Figure 4a. For the AFM coupling in Figure 4a, the upper-spin $e_{g}$ level of Mn ion couples with that of another Mn ion with the same level, the occupied $e_{g}$ levels are pushed up by Δ*d* energy, and the unoccupied ones are pushed down by Δ*d* energy due to the half-occupation of the $e_{g}$ levels, as shown in Figure 4b, thus, the super-exchange interaction is smaller than the double exchange interaction. By increasing distance between the two Mn^2+^ ions, the AFM coupling of d-d state becomes weaker, and the doped system can form paramagnetic or spin-glass states, which is consistent with our analysis about geometric structure and stability in previous section. The Mn-doped ZnO nanowires show an AFM behavior, and are prone to form a zigzag chain of Mn–O–Mn atoms along the [0001] direction, which indicate that the doped systems have a good effect on the [0001] preferred orientation to prepare ZnO-based dilute magnetic semiconductors.

**Figure 5:**
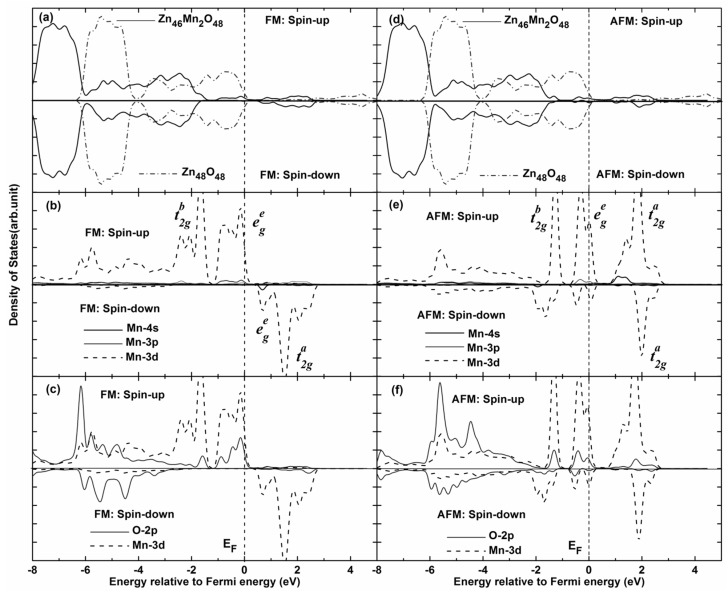
Total density of states (TDOS) and partial density of states (PDOS) of Mn-doped ZnO NW. (**a**) TDOS of pure Zn_46_O_48_ and Mn-doped ZnO nanowire for FM states; (**b**) PDOS of Mn atom for FM states; (**c**) PDOS of Mn 3*d* and O 2*p* for FM states; (**d**) TDOS of pure Zn_46_O_48_ and Mn-doped ZnO nanowire for FM states; (**e**) PDOS of Mn atom for AFM states; (**f**) PDOS of Mn 3*d* and O 2*p* for AFM states.

In order to further explain the magnetic coupling of Mn-doped ZnO NW, Figure 5 shows the total density of states (TDOS) and partial density of states (PDOS) of FM and AFM states. It can be seen that the TDOS of Mn-doped ZnO NW moves towards low energy (0.95 eV), and one can see that the spin-up Mn *3d* states pass through the Femi level for FM states, which confirms that the doped system is a half-metallic ferromagnet and exhibits 100% spin polarization. The strong FM coupling between the Mn *3d* and O *2p* states occurs at the top valence band (−1.0–0 eV) and the bottom valence bands (−4.0–−7.0 eV), the dispersive bonding $t_{2g}^{b}$ state and the local nonbonding $e_{g}^{e}$ state is formed at the valence band (−2–−6 eV) and near the Femi level, respectively. The antibonding $t_{2g}^{a}$ state is formed at the conduction band (1.6–2.1 eV). In particular, one can see from Figure 5c that the role of the O atom mediates the magnetic coupling between Mn atoms. The magnetic coupling chains of Mn^2+^–O^2^−–Mn^2+^ would reveal a double exchange mechanism owing to impurity states in the gap region. On the other hand, the magnetic moments of Mn atoms are 4.012–4.193 μ_B_ and mainly come from the Mn 3*d* orbitals, which are less than the theoretical value of 5 μ_B_. However, small contributions to the magnetic moments come from O 2*p* orbitals. As for AFM state, we find that the spin-up and spin-down DOS are asymmetrical, the spin-up electrons are more than the spin-down electrons. As the DOS of spin-up and spin-down states all pass through the Femi level, the AFM state is a possible metal ferromagnet. Spin-exchange splitting is also observed between Mn 3*d* and O 2*p* states, as shown in Figure 5e,f. Thus, the local bonding $t_{2g}^{b}$ states are fully occupied between −1.5 eV and −1.0 eV. Whereas the nonbonding $t_{2g}^{a}$ states are 4/5 occupied between −1.0 eV and the Femi level, the anti-bonding $e_{g}^{e}$ states are full empty between 0.6 eV and 2.5 eV, which correspond to the Mn *3d* states. We note that the bandwidth of the bonding and antibonding band is greatly narrowed for AFM states, and the spin-orbital coupling and hybridization effects are significantly higher than FM states. Therefore, stable AFM states can be easily formed in Mn-doped ZnO NWs. This is mainly because the Mn^2+^ (3*d*^5^) ion shows a half-full *3d* electronic configuration, the spin-up *3d* orbitals of Mn^2+^ atom are all occupied, and there are no excessive unoccupied orbitals for hybridization coupling with neighboring Mn^2+^ ions. As a result, *3d* orbital electrons of the neighboring Mn^2+^ are prone to filling the levels along the reverse parallel direction, which is in accordance with the mechanisms shown in Figure 3 and Figure 4. In addition, the up-spin or down-spin orbitals are fully empty for the higher $e_{2g}$ state and AFM coupling is favored. Namely, the AFM coupling can be formed more easily in Mn-doped ZnO NWs according to theoretical analysis, which is similar to the reported results [23,24].

**Figure 6:**
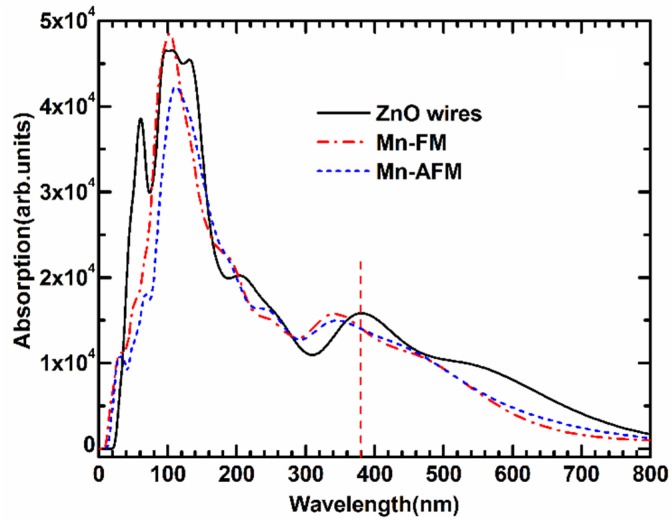
The optical absorption curves of FM and AFM for Mn-doped ZnO NW.

The optical absorption spectrum of Mn-doped ZnO NWs is illustrated in Figure 6. We applied the scissors operator (*E_scissor_* = 1.51 eV) to adjust the experimental and calculated band gap based on the experimental result (3.37 eV). To study the effect of Mn-doped on the optical properties, the absorption spectrum of pure ZnO NWs is also calculated. Some previous experimental studies [25,26,27] found that pure ZnO NW shows a strong absorption peak in the ultraviolet region as a result of near-band edge absorption. A wide green-yellow luminous bands form within the visible region of 550–600 nm. As shown in Figure 6, the calculated optical properties show that the absorption spectrum of the Mn-doped ZnO NWs are blue-shifted in the ultraviolet region, and the broad ultraviolet emission from 320 nm to 340 nm is observed in a doped system, which is related to electron transitions from the top valence band to the bottom conduction band. The calculated result is similar to the reported experimental optical absorption spectra of Mn-doped ZnO nanoparticles [28,29] and Mn-doped ZnO nanowires [30,31]. Additionally, one “sharp” absorption peak appears in the far-ultraviolet region near 90 nm, which are transitions from the *s*–*d* and *p*–*d* coupling of localized Mn 3*d* orbitals. The increase in the intensity of the UV peak is considered to be due to the participation of the 3*d* electrons of Mn [10,29,30], and is very similar to Co-doped ZnO nanowires [32]. The above-calculated results indicate a strong photo-response for ultraviolet-light luminescence. Namely, the Mn-doped ZnO NWs may be a new type of magneto-optical material with great promise.

## 4. Conclusions

In summary, geometric structures, and electronic and magnetic properties of Mn-doped ZnO NWs were investigated by using first-principles methods based on DFT. The magnetic origination and magnetic coupling mechanism were analyzed in detail. The results indicate that AFM coupling is the most stable for six doping configurations, and *p*–*d* hybridization coupling occurred at the top valence bands between Mn 3*d* and O 2*p* states. The dispersive bonding state and local nonbonding state are formed in valence bands, and a local antibonding state is formed in conduction bands. Magnetic moments are originated mainly from Mn 3*d* orbitals. The theoretical results further reveal the half-metallic magnetic properties of FM coupling and metallic magnetic properties of AFM coupling. Our research will provide theoretical guidance for preparing ZnO-based DMSs with a high Curie temperature.
